# Concurrent Umbilical Hernia Repair at the Time of Liver Transplantation: A Six-Year Experience from a Single Institution

**Published:** 2018-02-01

**Authors:** A. J. Perez, I. N. Haskins, A. S. Prabhu, D. M. Krpata, C. Tu, S. Rosenblatt, K. Hashimoto, T. Diago, B. Eghtesad, M. l. J. Rosen

**Affiliations:** 1 Comprehensive Hernia Center, Digestive Disease and Surgery Institute, The Cleveland Clinic Foundation, Cleveland, OH, USA; 2 Department of Quantitative Health Sciences, Lerner Research Institute, The Cleveland Clinic Foundation, Cleveland, OH, USA; 3 Transplantation Center, Digestive Disease and Surgery Institute, The Cleveland Clinic Foundation, Cleveland, OH, USA

**Keywords:** Cirrhosis, Clinical decision-making, Liver disease, Tissue injury and repair, Surgical technique, Umbilical hernia, Liver transplantation

## Abstract

**Background:**

Umbilical hernias are common in patients with end-stage liver disease undergoing liver transplantation. Management of those persisting at the time of liver transplantation is important to define.

**Objective:**

To evaluate the long-term results of patients undergoing simultaneous primary umbilical hernia repair (UHR) at the time of liver transplantation at a single institution.

**Methods:**

Retrospective chart review was performed on patients undergoing simultaneous UHR and liver transplantation from 2010 through 2016. 30-day morbidity and mortality outcomes and long-term hernia recurrence were investigated.

**Results:**

59 patients had primary UHR at the time of liver transplantation. All hernias were reducible with no overlying skin breakdown or leakage of ascites. 30-day morbidity and mortality included 5 (8%) superficial surgical site infections, 1 (2%) deep surgical site infection, and 7 (12%) organ space infections. Unrelated to the UHR, 10 (17%) patients had an unplanned return to the operating room, 16 (27%) were readmitted within 30 days of their index operation, and 1 (2%) patient died. With a mean follow-up of 21.8 months, 7 (18%) patients experienced an umbilical hernia recurrence.

**Conclusion:**

Despite the high perioperative morbidity associated with the transplant procedure, concurrent primary UHR resulted in an acceptable long-term recurrence rate with minimal associated morbidity.

## INTRODUCTION

Chronic liver disease (CLD) affects 4.5% to 9.5% of the general population worldwide [1]. Those patients with the most severe form of CLD, characterized by the presence of liver cirrhosis, portal hypertension, and ascites, are the patients who progress to liver transplantation. Of those patients being evaluated for liver transplantation, the incidence of umbilical hernia is estimated to be 20% [2]. To put this in perspective, it is estimated that less than 1% of the general population of the USA will develop an umbilical hernia in their lifetime [3]. The difference in the incidence of umbilical hernias between these patient populations is likely related to increased intra-abdominal pressure, weakening of the abdominal wall fascia, and muscle wasting due to poor nutritional status, and portal hypertension leading to a dilated umbilical vein enlarging the umbilical fascial opening in patients with CLD [2, 4]. Nevertheless, despite the relatively high incidence of umbilical hernias in the patient population with CLD, there remains a paucity of literature directing the management of this common problem in patients planning to undergo liver transplantation. 

Approximately 7000 liver transplantations were performed in the USA in 2015 [5]. It can be estimated that 1400 of those liver transplant patients will have developed an umbilical hernia by the time of liver transplantation. Repairing the umbilical hernia prior to the transplantation may increase the risk of ascites leakage, bleeding due to uncontrolled portal hypertension, mesh infections, and eventual delay of liver transplantation due to complications. However, if the hernia is not addressed at the time of transplantation, it could result in acute incarceration of the hernia contents, as a result of the rapid resolution of the ascites post-transplantation. Furthermore, the most appropriate approach to repairing an umbilical hernia during liver transplantation is complicated by the contamination inherent to the procedure and the burst of immunosuppression, both of which are felt to be relative contraindications for synthetic mesh placement. Most transplant surgeons, therefore, either avoid repairing these hernias at the time of transplantation or perform a primary repair at the time of transplantation.

The management of umbilical hernias in patients on the liver transplant list is controversial, with little published data to guide transplant and general surgeons. 

Our institution is a high-volume transplantation center with significant experience with this patient population. Our group prefers to perform a primary tissue repair of the umbilical hernia defect when present at the time of liver transplantation. The objective of this study was to evaluate 30-day morbidity and mortality outcomes and long-term hernia recurrence rates in those patients undergoing concurrent primary umbilical hernia repair (UHR) at the time of orthotopic liver transplantation at a single institution.

## PATIENTS AND METHODS

Consecutive adult patients undergoing primary UHR at the time of liver transplantation from January 2010 through June 2016 at the Cleveland Clinic Foundation were identified within a prospectively-maintained, institutional liver transplantation database. All umbilical hernias were repaired primarily via exposure from the Chevron incision used for the liver transplantation with the use of simple interrupted or figure-of-eight permanent sutures by the transplant surgeon without an additional incision over the site of the umbilical hernia. Patients undergoing only liver transplantation and those patients undergoing repair of abdominal wall hernias other than umbilical hernias, including ventral/incisional hernia repair or inguinal hernia repair, were excluded from this analysis. 

After obtaining Institutional Review Board approval, retrospective chart review was performed on all identified patients. Patient demographic information, operative details, 30-day post-operative outcomes, and long-term hernia recurrence outcomes were collected. At our institution, all patients undergoing liver transplantation are followed at the transplant clinic at one week, one month, three months, six months, nine months, and one year, post-operatively and then annually, thereafter. 

Disease severity was assessed by the Charlson Comorbidity Index, the Model for End-Stage Liver Disease (MELD) score, and the Sodium-Adjusted MELD Score [6-8]. These scores were calculated at the time the patients admitted to the hospital for liver transplantation to most accurately reflect their associated comorbidities and extent of CLD. Thirty-day outcomes of interest included the incidence of superficial surgical site infection (SSI), deep surgical site infections, organ space infections, wound dehiscence, unplanned return to the operating room (OR), unplanned readmission to the hospital, and death. Long-term outcomes of interest included hernia recurrence, additional abdominal surgeries, and death. Hernia recurrence was defined as a defect in the fascia at the site of the umbilicus either on physical examination or radiographic imaging at any of the above-mentioned follow-up appointments. Kaplan-Meier survival anlaysis was used to determine the length of time to hernia recurrence, taking into account those patients who were lost to follow-up and those who died. All statistical analyses were performed using SAS (*ver* 9.4, Cary, NC, USA).

## RESULTS

Approximately 135 patients are transplanted at our institution annually. From January 2010 through June 2016, 59 patients underwent primary UHR at the time of liver transplantation at our institution (Table 1). The majority of the patients were Caucasian (84%) and male (88%). With respect to the severity of illness, patients had an average Charlson Comorbidity Index of 5.2, a MELD Score of 18.1, and a Na-MELD Score of 21.3. Of note, 19 (32%) patients had hepatocellular carcinoma at the time of liver transplantation.

**Table 1:** Patients characteristics (n=59)

Variable	Mean±SD or n (%)
Age (yrs)	58.0±9.8
Sex	
Male	52 (88%)
Female	7 (12%)
Race	
Caucasian	48 (81%)
African-American	5 (9%)
Other	6 (10%)
Charlson Comorbidity Index Score	5.2±1.6
Age Adjusted Charlson Comorbidity Index	6.5±1.9
MELD Score	18.1±9.1
Na-MELD Score	21.3±8.6
Presence of HCC	19 (32%)
Presence of ascites	57 (97%)

In terms of 30-day morbidity and mortality outcomes, five (9%) patients experienced a superficial SSI, one (2%) patient experienced a deep SSI, and seven (12%) patients experienced an organ space infection (Table 2). Of the five patients who developed a superficial SSI, one was at the site of the umbilical hernia repair; this patient went on to develop an umbilical hernia recurrence. No deep or organ space infections occurred at the site of the UHR.

**Table 2:** 30-day outcomes (n=59)

Outcome	n (%)
Superficial SSI	5 (9%)
Deep SSI	1 (2%)
Organ space infection	7 (12%)
Wound dehiscence	1 (2%)
Unplanned return to the OR	10 (17%)
Unplanned hospital readmission	16 (27%)
Umbilical hernia recurrence	1 (2%)
30-day mortality	1 (2%)

SSI: Surgical site infection

Ten (17%) patients experienced an unplanned return to the OR; eight (80%) patients returned to the OR for coagulopathy-related intra-abdominal hemorrhage, one (10%) patient returned to the OR for repair of a biliary duct anastomotic leak, and one (10%) patient returned to the OR for emergent repair of an incarcerated inguinal hernia. Sixteen (27%) patients had an unplanned readmission to the hospital, all of which were unrelated to the UHR, and one (2%) patient died within 30 days of their index procedure due to multiorgan system failure following a biliary anastomotic leak. 

With respect to long-term outcomes, patients were followed for a mean of 21.8 months. At the time of chart review, there were data available for 39 (66%) patients. Of the original 59 patients, there were nine (15%) patient deaths and 11 (28%) patients who were lost to follow-up. Seven (18%) patients had an umbilical hernia recurrence diagnosed by either physical examination (n=5) or radiographic imaging (n=2). The mean±SD time to recurrence was 4.3±2.5 months. The aforementioned UHR recurrence after superficial SSI was the only one of the seven recurrences to have developed a post-operative infection of any kind. Of the seven umbilical hernia recurrences, three patients underwent elective recurrent hernia repair and one had urgent primary repair for incarceration. Two patients had primary suture repair of their recurrent hernia, one was repaired using an open retrorectus placement of synthetic mesh, and another had an onlay repair of their umbilical hernia with Permacol mesh concurrently during primary repair of bilateral subcostal incisional hernias and a roux-en-y hepaticojejunostomy for biliary stricture.

**Figure 1:**
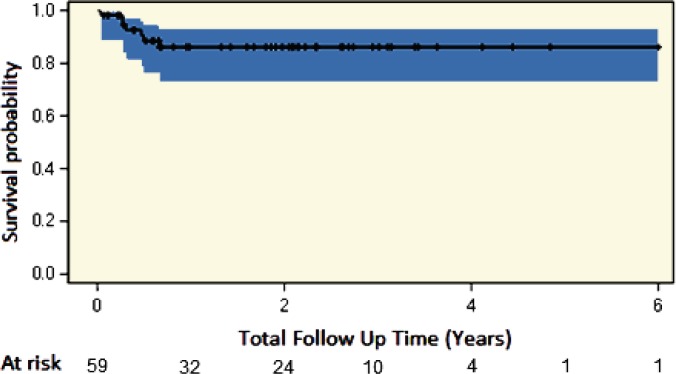
Hernia recurrence free survival estimate with number of subjects at risk

Due to the inherent death and loss to follow-up over time, a Kaplan-Meier analysis was performed to determine the rate of hernia recurrence-free survival over time. Umbilical hernia recurrence was noted to occur early (within six months) following primary UHR at the time of transplantation (Fig 1).

## DISCUSSION

The timing of UHR in patients with end-stage liver disease gives pause to many surgeons due to the inherent risk and comorbidities of the patient. Some surgeons choose to undertake an elective repair prior to transplantation; others choose a conservative “watch-and-see” approach with repair at time of symptom development, whether it is before or after transplantation, while others choose elective repair at the time of liver transplantation. In a retrospective review of 34 patients, Marsmen, et al, compared the outcomes of umbilical hernia management in patients with liver cirrhosis and ascites. Due to the high incidence of morbidity and mortality noted with conservative “watch-and-see” treatment, they advocated that all umbilical hernias be repaired electively prior to transplantation, if possible [2]. Despite this recommendation, there still remain patients with persistent umbilical hernias at the time of transplantation. Our findings suggest that umbilical hernias that do remain present at the time of transplantation can be primarily repaired at the time of orthotopic liver transplantation with minimal increased morbidity and with acceptable long-term results [9-12].

For persistent umbilical hernias, the risk of incarceration and strangulation has been observed to be the highest following liver transplantation due to the resolution of ascites and associated fluid shifts, necessitating emergency surgery shortly after liver transplantation [11]. Furthermore, the presence of incarceration or strangulation subjects the skin overlying the umbilical hernia to thinning, infection, and even rupture [13]. Therefore, with primary tissue repair of the umbilical hernia at the time of liver transplantation, the hernia can be fixed, if not, temporized so that the risk of incarceration, strangulation, and emergency surgery minimized. In the event of a recurrence, a more complex hernia repair with the use of mesh can be pursued following improvement in liver-associated impairments, including coagulopathy and malnutrition.

Our results demonstrated that repair of the umbilical hernia from within the abdomen using the same transplantation incision added very little to the operation time or the overall morbidity of the liver transplant surgery. Furthermore, we found that the long-term hernia recurrence rate in our series was 18%—a much more acceptable rate than that previously described by de Goede [14]. In this retrospective review of 27 patients who underwent concurrent UHR, either through a separate incision or the same transplant incision, at the time of liver transplantation, there was an alarming 40% recurrence rate with a 50% complication rate; this was however among a very small sample size of 10 patients undergoing same incision UHR [14]. 

Compared to the long-term umbilical hernia recurrence rates of 8%–54% mentioned for the general population, our recurrence rate of 18% was acceptable [15]. Our recurrence rate, which approached that of the general population, was a bit surprising given the considerable predisposing comorbidities for hernia recurrence in this patient population, including poor wound healing and coagulopathy in the setting of immunosuppression, impaired hepatic synthetic function, and malnutrition found among patients undergoing liver transplantation. The relatively low recurrence rate may be explained by the resolution of ascites and improvement in liver function after liver transplantation which likely plays a role in the durability of these UHRs due to the contraction of the tissues, decreased straining forces on the repair from the resolution of intra-abdominal ascites, and improvement in nutritional and wound healing parameters. Possibly, the “tissue expansion” resulting from the increased intra-abdominal pressure caused by the ascites prior to the transplantation may counterbalance the acute tension on the primary repair. Therefore, we support the concurrent primary repair of all umbilical hernias present at the time of liver transplantation. 

Despite our results, our study did have limitations that are worth mentioning. First, this was a retrospective study with all of its inherent weaknesses. Second, this study was performed at a quaternary referral center. While this allowed for significant long-term follow-up, these results may not be reproducible amongst all centers performing liver transplantation. Finally, hernia-specific data including hernia size were not available for analysis. In the absence of these data, we were unable to comment specifically on the management of umbilical hernias with regard to the defect size.

In conclusion, we found that despite the high perioperative morbidity associated with the index transplant procedure, the long-term results of primary UHR from within the abdominal cavity without the use of a separate incision resulted in acceptable wound and hernia-related outcomes at our institution. These findings suggested that primary repair of asymptomatic umbilical hernias at the time of liver transplantation would be a safe and effective approach. Further hernia-specific data are required to determine patient populations that may benefit from alternative management strategies. 
